# Caught between a rock and a hard place: mental health of migrant live-in caregivers in Canada

**DOI:** 10.1186/s12889-017-4431-4

**Published:** 2017-05-23

**Authors:** Mandana Vahabi, Josephine Pui-Hing Wong

**Affiliations:** 1 Daphne Cockwell School of Nursing, Faculty of Community Services, Ryerson University, Toronto, Canada; 2 Daphne Cockwell School of Nursing, Faculty of Community Services Ryerson University, Toronto, ON Canada; 3 Ryerson University, 350 Victoria Street, Toronto, ON M5B 2K3 Canada; 4 Ryerson Centre for Global Health and Health Equity, Toronto, ON Canada

**Keywords:** Temporary foreign workers, Live-in caregivers, Low skilled workers, Mental health, Social exclusion, Otherness, Social identity, Public versus private transcript

## Abstract

**Background:**

Canada depends on Temporary Foreign Workers (TFWs), also known as migrant workers, to fill labour shortage in agriculture, hospitality, construction, child/senior care, and other low-skilled occupations. Evidence shows that TFWs, especially women live-in caregivers (LC), constitute a vulnerable population. Their health is compromised by the precarious and harsh working and living conditions they encounter. There is a paucity of research on the mental health of LCs, their support systems and access to mental health services.

**Method:**

In this community-based exploratory study, we used mixed methods of survey and focus groups to explore the work related experiences and mental health of migrant live-in caregivers in the Greater Toronto Area in Ontario, Canada. Convenience and snowball sampling were used to recruit participants. The inclusion criteria were: being 18 years or older, initially migrated to Canada as TFWs under LC program, resided in the Greater Toronto Area, and able to understand and converse in English based on self-report. This paper reports on the focus group results derived from inductive thematic analysis.

**Results:**

A total of 30 women LCs participated in the study. Most of them were from the Philippines. A number of key themes emerged from the participants’ narratives: (1) precarious migration-employment status (re)produces exploitation; (2) deskilling and downward social mobility reinforce alienation; (3) endurance of hardship for family back home; (4) double lives of public cheerfulness and private anguish; and (4) unrecognized mental health needs. The study results reflected gross injustices experienced by these women.

**Conclusion:**

A multi-faceted approach is required to improve the working and living conditions of this vulnerable group and ultimately their health outcomes. We recommend the following: government inspection to ensure employer compliance with the labour standards and provision of safe working and living conditions; change immigration policy to allow migrant caregivers to apply for permanent residence upon arrival; the TFWs Program to establish fair wages and subsidized housing so that caregivers can truly access the live-out option; and local ethno-specific, settlement and faith organizations be leveraged to provide TFWs with social support as well as information about their rights and how to access health and social care.

## Background

Canada has relied on temporary migrant labour for its economic prosperity for more than five decades. Canadian employees recruit and hire temporary foreign workers (TFWs) to fill the labor gaps in both skilled and non-skilled trades. Although TFWs play a significant role in Canada’s economy, they have remained as an invisible and underpaid workforce. TFWs often work in substandard working conditions that most Canadians do not accept or are protected from because of different sets of labour laws and regulations that govern permanent residents and citizens versus temporary migrant workers. The precarious employment and migration status render TFWs a vulnerable group.

The Temporary Foreign Worker Program (TFWP) includes numerous programs - Seasonal Agricultural Worker Program, Caregiver Program (formerly known as the Live-in Caregiver Program (LCP), and Low and High Skilled Program. These programs are regulated by the Human Resources and Skills Development Canada (HRSDC) which defines the types of jobs to be filled by TFWs, the numbers of workers to be admitted each year, and policies and terms around their employment [1]. Between 2011 and 2014, the number of work-permit holding temporary residents in Canada exceeded the number of new permanent residents with a peak number of close to 310,000 in 2013. The number has decreased to over 250,000 in 2015 but they still made up of close to 48% of newcomers in Canada [2]. The main source countries for migrant workers include the Philippines, the Caribbean, Mexico, Guatemala and India [3]. Alberta, British Columbia, Ontario, and Quebec receive the majority (91%) of these workers [4].

The Live-in Caregiver Program (LCP) was introduced in 1992 in Canada; currently, it consists of two components: the Caring for Children Pathway and the Caring for People with High Medical Needs Pathway. It recruits qualified temporary foreign workers to come to Canada to care for children, older individuals and people with disabilities in their homes [5, 6]. The LCP has taken on more prominence given that Canadians lack access to universal affordable childcare, and the Canadian health and social care systems are not meeting the emerging needs of Canada’s aging population despite the current discourse of maintaining elderly people at home for as long as possible [7, 8]. The LCP attracts highly qualified migrant workers because it allows caregivers to apply for permanent residence in Canada after they have completed 24 months of work as caregivers [5]. In 2014, there were 16,238 TFWs in Canada with a valid permit for Caregivers [4].

Prior to 2014 the LCP required the caregivers to live with their employers, placing them in vulnerable situations such as working long hours of uncompensated overtime, receiving low pay due to deductions by employer as room and board, having no private space and time, and separation from their families for long periods of time [8–12]. Over the past few years, the Canadian government had brought in changes that aimed to improve the program. In November 2014 the requirement of the caregiver to live within the employer’s residence was removed [6]. Also, employers of low-wage live-in caregivers must provide accommodation that meets municipal building codes, and a private and furnished bedroom with a lock and safety bolt on the inside [13]. However, the Canadian government had also imposed higher levels of language and education requirements for TFWs who are applying to become permanent residents after they have fulfilled their 24 months of work as live-in or live-out caregivers [5, 6]. As these changes are relatively recent, little is known about their effects on the health and quality of life of temporary migrant caregivers.

### Health of temporary foreign workers

There is a paucity of research on the health of TFWs, especially on temporary live-in caregivers who are mostly women. Within the sparse literature on live-in caregivers in Canada, most of the studies focused on the phenomena of global care chain, jurisdiction boundaries, deskilling of professional trained women, managed immigration and policy analyses [8, 14–16]. Similarly, research on the broader TFW population in Canada and the US has also focused mostly on labour issues and occupational health and safety of migrant farm workers employed through the Seasonal Agricultural Workers Program (SAWP) and these workers were mostly from Mexico and the Caribbean [17]. Despite substantial knowledge gaps, the limited literature on the health of TFWs does provide an overview of the health challenges experienced by TFWs and the associated factors that shape their health. Research evidence shows that many TFW have experienced the *healthy immigrant effect,* i.e., TFWs arrived Canada with excellent health (a requirement for immigration and work permits) but experienced progressive health decline as they worked in Canada. This health decline was found to be associated with interpersonal and structural challenges specific to their jobs, including a lack of freedom to choose or change employers, unsafe working conditions (e.g., long work hours, lack of job safety training, and repetitive injuries), substandard living conditions, (e.g., overcrowding, poor housing, and limited access to healthy food) and fear of deportation [17–20]. Many TFWs developed chronic and infectious diseases such as HIV and TB [19–21]. The high use of alcohol and drug as a coping strategy, particularly among migrant men, was also reported [22].

Although employers of TFWs are required to provide health care insurance coverage for their employees, many TFWs have limited access to health care due to the bureaucratic challenges and delays they face in obtaining their health cards [23]. Many do not seek health care because of a lack of familiarity with Canadian health care system, limited knowledge about their rights in accessing health care, working long hours which go beyond the traditional hours of medical and social services, and having language and cultural barriers [17]. Furthermore, many TFWs do not openly acknowledge their injuries or health problems because of their fear of repatriation -- being sick or seeking medical care may put their livelihood at jeopardy as the TFW programs only admit and keep healthy migration workers [23, 24]. Many TFWs resort to self-medication, which has been reported to be a common practice among this vulnerable population [25]. The myriad systemic inequalities experience by TFWs in their everyday life, in the form of racism, unfair employment practices, precarious migration status, separation from family and friends, lack of social support, and sense of powerlessness and isolation often lead to depression, anxiety and stress [23, 24, 26]. However, little is known about LICs’ mental health and their experience in accessing mental health care. In fact, few studies have focused on the health of women migrant live-in caregivers, whose everyday challenges differ from the other TFWs. To address this gap, we undertook an exploratory study to gain a better understanding of the work related experiences and mental health of migrant live-in caregivers in the Greater Toronto Area in Ontario, Canada.

## Methods

This exploratory study is guided by a Population Health Promotion Framework grounded in the principles of social justice an equity and use of a socio-environmental approach to identify the contexts and conditions that contribute to health disparities and strategies to promote health of marginalized and vulnerable populations [27–29]. Explicit in this framework is the assumption that health disparities are produced by myriad social determinants including access to: adequate income, employment and housing; physical, psychological, social and financial security; and stress associated with experience of social exclusion. In this study, we recognize that the life circumstances of live-in caregivers are shaped by systemic exploitation issues in both Canada and their countries of origin. We used mixed methods (surveys and focus groups) to explore: 1) migrant live-in caregivers’ job related experience and mental health; 2) their perceived barriers and facilitators in accessing mental health services, and 3) the individual and structural determinants of their mental health. Ethics approval for the study protocol was received from the Research Ethics Review Board at Ryerson University (REB 2014–175) and informed consent was obtained from each participant. Each participant received a $40 honorarium for their participation. Data were collected through the use of a detailed questionnaire followed by focus groups. This paper focuses on the findings from the focus group component of the study.

### Sample

We used purposive sampling to engage live-in caregivers. Participation criteria include: aged 18 years or older, residing in the Greater Toronto Area, temporarily migrated to Canada within the past 6 years (i.e., 2008–2014) initially through the Live-in Caregiver program (now known as the Caregiver Program), and were able to understand and converse in English based on self-report. A peer research assistant, who used to be a live-in caregiver, was hired to help with recruitment. Participants were recruited either through community-based organizations working with migrant populations in Greater Toronto Area and through snow-ball technique by asking potential participants interested in this study to refer other live-in caregivers to this study.

### Data collection and analysis

Data collection occurred between December 2014 to February 2015 through self-completed questionnaires and focus groups. Three semi-structured focus groups were conducted by one of the researchers (MV) to explore participants’ everyday experience, including their: 1) working and living conditions; 2) beliefs and perceptions about what contributes to one’s mental health;, 3) challenges and facilitators in maintaining one’s mental health; and 4) suggestions for inclusive and relevant strategies to promote mental well-being of live-in caregivers. Within the semi-structured focus group approach, participants were encouraged to express their perspectives and described their experiences beyond the restriction of the focus group question guide but at the same time engage in dialogue with a specific focus on the study topic. Each focus group consisted of 10 participants and lasted about 2 h. It was conducted in English and was audio-recorded with the permission of the participants. The interview guide included 3 major questions:

1. *What are the experiences of temporary foreign workers (TFW) living and working in Canada?*
2. *What are your beliefs and values about mental health?*
3. *What are the mental health issues faced by TFW living and working in Canada?*

The audio-taped focus groups were transcribed verbatim, producing 132 pages of data. We used an inductive interpretive approach [30] to guide the thematic analysis of the data through: (1) repeated reading of the transcripts to develop a broad and familiar understanding of the data; (2) detailed line-by-line coding of key concepts and ideas that were articulated by the participants, themes that were specific to our research questions, and sensitizing concepts based on our theoretical concepts/frameworks [31]. This form of thematic analysis, or pattern coding, enabled the researchers (MV and JW) to review each focus group transcript independently to identify themes and organize voluminous data into smaller and meaningful analytic units [32]; it also enhances the researchers’ ability to engage in open dialogue about the identified themes and collectively engaged in data interpretation to ensure rigour, auditability and trustworthiness. Integrity of data interpretation was maintained following strategies of credibility (peer debriefing) and auditability.

## Results

### Participants

A total of 30 women migrant live-in caregivers took part in this study. Twenty-five of them (83%) were Filipina and five (17%) were Eastern European (i.e., from Hungary, Ukraine, and Poland). The average age of respondents was about 41 years, ranging from 25 to 60 years. Slightly over half of the participants were married. Half of the participants reported having children and of those who had children, 67% had one or two children and 73% did not have their children living with them in Canada. The majority of participants (83%) had either some or completed college or university training and overall participants reported high English literacy. Eighty percent of the women had been in Canada for more than 2 years. The majority of respondents (83%) were employed full-time but a considerable portion lived below the Canadian after tax income cut-off point (73% reported an average after tax monthly income of less than $1999 and of those 10% had an income of less than $1000). The majority of participants (i.e. 28) worked as live-in caregivers but two participants who initially migrated to Canada under LC program have changed to work in food services. More than half of the participants (63%) worked more than 40 h per week. Ninety percent lived with their employers either in an apartment (56.7%) or in a house (33.3%). Half of the participants shared their bedroom with other people. Of those, 93% shared with at least 1–2 people. Table 1 provides the study participant’s socio-demographics.

**Table 1:** Socio-demographic Characteristics of Participants

	#	%
25–34 years	12	40.0%
35–49 years	11	36.7%
50–60 years	7	23.3%
Gender		
Female	30	100%
Place of Birth		
Philippines	25	83.3%
Other (i.e. Hungary, Ukraine, Poland)	5	16.7%
Length in Canada		
Less than one year	3	10.0%
12–24 Months	3	10.0%
More than 2 years (more than two years)	24	80.0%
Current relationship status		
Single, Never married	10	33.3%
Married	16	53.3%
Separated/Divorced	4	13.3%
Do you have children		
Yes	15	50.0%
No	15	50.0%
Number of children		
1	3	20.0%
2	7	46.7%
3 or more	5	33.3%
Number of children living with you in Canada		
None	11	73.3%
0–2	4	26.7%
Highest level of education attained		
High School (12 grades) some or completed	2	6.7%
Other vocational training some or completed	1	3.3%
College (e.g. diploma) some or complete	17	56.7%
University (e.g. BA, BSc) some or completed	8	26.7%
Post- graduation (e.g. MA, PhD) some or completed	2	6.7%
Total	30	100.0%

### Working-and-living conditions

For most of the participants, their working and living conditions were intricately intertwined – they worked and lived inside their employers’ homes where their work life and personal life were blurred in the contexts of time and space. They experienced myriads of overlapping demands and conditions that created stress and compromised their mental health.

#### Substandard working conditions

Out of 30 participants, only three spoke positively about their working conditions; these three live-in caregivers considered themselves to be blessed to have good employers, which suggested that it was not the norm. Their so-called blessing premised on the employers following the rules and agreement of working conditions in the signed employment contract.

*In my case I’m so blessed that I’ve had good employers and I only work specific time, eight hours and after that I have my own free time so after my work I will do my studies…* Participant from Focus group 2

The majority of participants reported that they worked in substandard conditions. Some suggested that working conditions for Live-In Caregivers in Canada were worse than those in other countries. Those who had worked in countries like Saudi Arabia prior to coming to Canada indicated that their working and living conditions (accommodation, amenities, treatment, and help with living expenses) in these countries were much better and respectful of their qualifications and credentials.

“*I went out of the country [the Philippines] in 1985, going to the Middle East countries and I think it’s better because once you start a job you can be promoted. I was there for 10 years. I was a nurse there and I was promoted. You know, once you have a contract you are assured that you have housing, you even have a car that will bring you from the workplace and bring you home. Not here”.* Participant from Focus group 3

In Saudi Arabia, nurses from the Philippines were hired as nurses and respected as professionals; they were not deskilled and hired as low-pay caregivers in substandard working conditions as they have been in Canada. For instance, most of our participants reported working long hours and performing tasks beyond what was stated in their employment contract.

*“In the contract it just says eight hours but no, it is 24 hours; we do laundry, cooking, cleaning which is not what we were told before [we came]. I go to work at 6.00 a.m. and finish my work at 10:30-11:00 p.m. I had many employers but all of them are the same. You are supposed to have two days off but no I have to work Saturdays and then I can go for a day and then come back again.”* Participant from Focus group 3

The long working hours without pay applied not only to live-in caregivers but also live-out caregivers. Furthermore, when the employers discovered their former education and expertise, they were expected to also apply these skills in their work but without fair compensation.

*“You sign off as a caregiver and your job description is only to look after the kids and doing some work related with their care. Now once you are there they will ask you to cook some dishes for them and they find you are a good cook they will keep on asking for that. That means that’s another add-on to your job description. If you are a teacher back home of course you know how to teach kids—that’s another add on to the job. If you are good in mending clothes again that’s another add on and it goes on and on. So work never stops.”* Participant from Focus group 3

Most participants indicated that the signed contract was only a piece of paper required for them to stay in Canada; their employers seldom honoured the terms and conditions stated in it.

Another work related challenge faced by some caregivers was the constant surveillance they experienced during their work. A few participants reported that they were being monitored by hidden cameras installed throughout their employers’ homes without their prior knowledge or permission. The constant surveillance imposed a great deal of mental health stress on the caregivers.

*“My house, the family that I live with, it’s stuffed with cameras…it is not like you do something bad to the kids but you have to all the time think about what you are doing—maybe they are not watching me but I think maybe they were; like my self-esteem goes very down and you feel, okay, no I’m not gonna do this thing because may be someone’s watching me”.* Participant from Focus group 2

Some participants described other stressors related to emotional and physical abuse they experienced on the job and their sense powerlessness to respond or stop the abuse*.*

*“My employer yells at me and the kids hit me. You cannot do anything, you cannot do anything. If you yell at them you get fired.”* Participant from Focus group 1

*“I was verbally abused by my client [employer]. They thought that we don’t know anything. Then I showed him my credentials, you have to know who I am and please do not talk to me like that. I was crying every night”.* Participant from Focus group 2

For some participants, having the courage and skills to communicate assertively to their employers had led to improved working conditions, but most endured in silence.

#### Being “captive labourers”

Some participants described themselves as “*captive labourer”* or “*prisoners”* who did not have any control over their work and living situations. Many felt isolated and powerless because they were aware of the unequal power relations between their employers and themselves.

*“Even if we know what our rights are, we might as well keep on doing our work for [the] 24 months; otherwise, if we complain work will stop, that will [be an] impedim[ent] to [completing] our 24 months. So we stay quiet.”* Participant from Focus group 2

Although participants were aware of their legal rights on paper, their fear of job termination was a major reason for not confronting their employers about the exploitative working conditions and the emotional and physical abuse.

*“You want your family to come as soon as possible, so after we finish the 24 months then we can start the paper work permit, and then permanent resident, and that will make it a little bit closer to the final point when they come, the reunification is what we’re looking forward [to]”.* Participant from Focus group 2

The participants’ desire to bring their families and children to Canada for reunification was a key force that kept them going despite the ongoing challenges in their caregiving jobs. They feared that any confrontation with their employers would lead to termination of their job. Since their work permits were tied to their existing jobs, finding new employment would be difficult, especially when they had to rely on their former employers to provide job references. Furthermore, job loss might lead to repatriation, delayed completion of the 24-month caregiving period required for their application to become permanent residents, and subsequent prolonged separation from their loved ones.

Others shared that they were treated like an object to be shared and used at the whim of their employers without being consulted first.

*“They share you [as a servant] with whoever they feel like… You think you are just working for this employer but it does not happen. I work also for the [employer’s] sisters and friends. You have to do it and you cannot say no”.* Participant from Focus group 2

One participant described the TFW program as the *“resurrection of slavery in 21st century”* because of her experience of abuse and exploitation by her employers and her sense of powerlessness to resist.

#### Housed but homeless

It was ironic that most of our participants worked inside the homes of their employers, carried out long hours of “domestic” work and yet did not really have a home of their own. Most of the participants were relegated to sleep in the basements that were cold and damp with dim lighting and poor air circulation. Some basements were not adequately furnished.

*“Even though the house is beautiful and they have many rooms and the rooms that they do not use have beautiful furniture, but whatever is left for the live-in caregiver is a basement room, windowless, airless, cold and damp.”* Participant from Focus group 1

Some also spoke about the lack of privacy and security in the living space provided by their employers.

*“I live with my employer, with no lock, just in the basement and a bed in there, but no lock, no room. I did not know that the bed that I sleep in was their dog’s bed until their daughter told me”.* Participant from Focus group 2

Others shared that the lack of privacy was not only pertaining to their physical living space, but also about the lack of psychological space and social boundaries for them.

*“Like when you live with people, you live with their lives, you know. You’re lucky if a family doesn’t have any problems… but many families they have inside some problems but they will not show outside, and basically you sometimes have to listen to them arguing or some other stuff and … You still hear them.”* Participant from Focus group 1

*“Or the children, they come and knock at your door and say, “Are you here?” You can’t reject them… like even though you love them, right, but you still … but at the same time you kind of feel - Okay, maybe I need to do something.”* Participant from Focus group 2

The participants’ narratives suggested that many of them experienced a phenomenon of what might be called “housed but homeless” (Anucha, [33]); they did not have access to any physical or psychological space where they could feel safe, secured and free to be who they were.

#### Caught between a rock and a hard place

When asked about their everyday experiences as caregivers in Canada, most of the participants spoke simultaneously about their despairs and determination to keep working. Their despairs were associated with their downward social mobility, precarious migration status and harsh working-living conditions after coming to Canada as migrant caregivers. Their determination to keep their jobs, despite all the hardship, was motivated by the demands of remittance to their home countries to support their families and relatives. Many reported that their professional credentials and skills were not recognized by Canada; their experience of being deskilled through their jobs as caregivers took a toll on their self-esteem.

“*We studied back home and then we did work as nurses, we did work as teachers. Basically that’s the main problem - here we are cleaning their houses and it’s hard for us to accept that.”* Participant from Focus group 2

*“Because we came here as a live-in caregiver I think they see us as lower. They don’t see us as well educated professionals but as maids.”* Participant from Focus group 2

The misperception and stereotypical beliefs about migrant caregivers as low-skill uneducated workers seemed to affect how some of the participants were treated by their employers.

*“I was a nurse. When I first came to Canada I was not expecting that the work of a caregiver was a maid… But when my agency brought me to my employer and he said to me, “You have to clean the house.”… I showed my credentials to him, “You have to know who I am, and please don’t talk like that. I’m going to work with your mom and your dad, I would really like to work with them.” Because this is what we need to do to finish these 24 months of employment so we can get the benefit of it, I was crying every night. I can’t tell it to my children because I don’t like my family to get worried.”* Participant from Focus group 2

Furthermore, many participants could not share their experiences of deskilling and despair with their families for the fear of causing them distress and pain. Some worried about breaking the rosy image of being a migrant caregiver in Canada.

*“I would never ever tell my mom what I’m doing here, it would just hurt her and she would buy me the next ticket back to Ukraine. Like I had a teaching job, right, I had a teacher’s diploma and I came here and I started to suddenly clean toilets and change diapers, and pick up the laundry that the woman drops on the floor… and I don’t want to tell this to my family… they cannot do nothing.”* Participant from Focus group 1

Despite having to face many challenges related to their perilous working conditions, many participants were determined to continue because of their desire and the pressure to support their immediate and extended families.

*“[Being a caregiver] it’s sometimes good, sometimes bad. There’s a lot of pressure. Like, you know, our family depends on us back home so of course financially we could send money … of course at the end … we need to meet both ends. At the end of the month or payday our … most of our pay we send back home.”* Participant from Focus group 3

The pressure to send money back home was especially intense for migrant caregivers whose loved ones were ill but they could not be by their side to provide care.

*Like in my case, my sister got a surgery and my mom was in ICU many times… so the only thing I can help is to send money, but your heart is just like … your [heart] got broken every time you get news. Say Monday and Wednesday it’s gone, money is gone, so it’s hard. Where can you get this loan? And then, you know, it’s so hard to meet both ends.* Participant from Focus group 3

Amidst their despair, many participants focused on their ultimate goal of getting permanent residency after their completion of 24 months of employment as caregivers.

*After I finish my contract what is the next step? I keep on thinking about all that and sometimes there are nights that I can’t sleep, I keep on crying... I really want to help [family] that’s why I need to do something. I have to save some money for my school. Still I keep on waiting for my PR* [Permanent Residency]*. If it comes then I can go for my schooling.* Participant from Focus group 2

Indeed, one participant shared that her endurance of the harsh working-living conditions did lead to her goal of reuniting with her family.

*I want my family to be here so I did sacrifice and it’s all worth it. So after my five years my daughter came and I was a live-out. I said [to employer], “I want to be a live-out now and the pay should be different.” […] You see, I am driving, I am cooking, a lot of cooking… So I said, “Cooking is a different pay…” And she said, “How much?” I want $20 per hour, that’s the cheapest I could give… And she said, ‘Oh you’re a good negotiator.” And this is what I did… my relationship with my employer is really very good.* Participant from Focus group 2

It was apparent that this participant’s assertiveness and confidence to negotiate for fair wages was underpinned by her legal right as a permanent resident.

### Stress, health decline and social support

When participants were asked to describe their current health, the theme of declining health since their arrival to Canada emerged. Many expressed worries about their general health problems; they noticed their bodies were reacting negatively to the ongoing stress they faced every day.

#### Stress related to work demands

Some participants suggested that the demanding and stressful working-living conditions had taken a toll on their health. They talked about the long hours that they worked every day. Most of them reported having four to 6 h of sleep each night, and they went to bed feeling exhausted, as one participant shared, “*Not only looking after the kids but you need to clean the house, shopping, cooking, cleaning, everything. You manage the whole house for the whole day. Physically, mentally, it’s all packed with stress.”* Participant from Focus group 2.

Another participant reflected on her health while working as a caregiver in Hong Kong and before she came to Canada:

*“So our blood pressure goes up, our blood sugar goes up, so to sum it up we came here healthy. I worked in Hong Kong 15 years and thank God I was okay. I did not have a cold, I didn’t have a flu or whatever, but when I came here there’s tons of it, one after another, our body deteriorates”.* Participant from Focus group 3

One participant suggested that her cancer was likely brought on by the demands of her job, *“I was diagnosed with having cancer and pretty much they think it’s at stage 4… Maybe I can say I got this sickness through my lifestyle, like working so hard, the stress is the number one factor why I got it”.* Participant from Focus group 3.

#### Stress related to loss and grief

In addition to the physical demands of their job, many participants described the emotional and mental health stress they experienced because of their precarious employment.

*“I am always worrying about my family… I am so stressed and actually I was on sick leave for 12 days because of the stress but what I keep worrying is about my family, that if I lose my job right now, because I know I’m not happy with it… If I leave this job and I cannot find a job tomorrow then I lose pay. It’s always like that. So instead of leaving the family that I’m working now which I am not happy, I have to stay which is hard. I have to stay… I’m worried my family because I’m earning the money for my family, not just for myself.* Participant from Focus group 2

They also talked about their experiences of stress, loss, and grief related to their separation from their families and loved ones.

*It happened to me last year, My father passed away and when I take care of papa [employer], I always think about my father – when he needed me most I wasn’t there but… what am I doing to… And last night I dreamed about my father and I ended up crying and I say, why is it that I dream about my father when he had already passed away?* Participant from Focus group 2

In addition to grieving the loss of their families and loved ones back home, some participants also lamented about the loss of a sense of closeness with their families after long periods of separation.

*I remember when my grandfather died in Poland I was so depressed, I cried. I remember now my grandmother died and she was very close to me… and I wasn’t there, I wasn’t there at the funeral… I feel bad about it sometimes because when I go there, they’re all together very close, you know, my niece - she’s more close with my brother’s girlfriend than with me, you know, because they just don’t know me. So sometimes it’s painful for me kind of stuff, you know, yeah.* Participant from Focus group 1

While the participants did not articulate their experiences of stress as mental health problems, many of them reported insomnia and sadness, as one participant shared, *“There are nights that I cannot sleep, [I] keep on crying.”* Participant from Focus group 2.

#### Ambiguous role of family as social support

When asked about what types of social supports they had, most participants shared that they mainly relied on friends and family. They also expressed a mistrust of strangers. However, although most of them identified family as their key social support, many also struggled about seeking support from their families. As described earlier, some of them expressed a *sense of shame and reluctance* to disclose the desolation, hardship and downward social mobility they faced in Canada. They wanted to protect their families against the pain and anguish that their stories might bring.

*I don’t tell anything my family, especially if my employer do something and I feel hurt and then I get a call from my mom or sister-in-law, “Oh, how are you?” And even though I don’t tell anything, “Oh I’m okay.” But the way you talk, your voice, they know that you’re hiding something. So Saturday night, oh I feel bad because my employer yelled at me… and the kids hit me so…* Participant from Focus group 3

As a result, many participants convinced their families and loved ones that their life in Canada was fine. They tried to cope with their stress and suffering on their own and this internalizing of suffering led to depressive symptoms such as crying every night.

“I feel lonely, very lonely. Lonely because I do not tell my family anything and do not want to talk to strangers because I’m thinking they may just mislead me”.

*“I just kept it to myself and just cry every night”.* Participant from Focus group 2

#### Social support beyond family

Most participants identified friends as an important source of support. Getting advice from friends was particularly important to caregivers who were new to Canada and were not aware of community resources.

*Well so far I didn’t need help but I didn’t know any places and yeah, like what if I [need help], the only place I will go to… will be my friends. These two ladies, they are my family. I will go to them first, the first place I will go to is them, like I know I can rely on them, but I mean services from the government or something, no, I don’t know.* Participant from Focus group 3

Many found comfort in discussing their issues with friends who shared similar experiences and understood their challenges. While they sought advice from their friends, they also recognized that their friends might be in similar situations, i.e., being unaware of their rights and available resources or options*.*

*“It is good to have friends, friends are friends, but they are the same as I am. They don’t know their rights, they do not know what to do in this situation …. I will ask for suggestions and they will be like, yeah, you should go here or may be, I don’t know there”.* Participant from Focus group 2

For those who had not established a network of friends, faith organizations became an important source of support, as one participant shared, *“I don’t have a lot of friends because I just work and work and I don’t have time to go out. I only go to church every Sunday.”* Participant from Focus group 2. Others found strength in connecting to their spirituality, “*For me personally, I’m Christian and I’m a religious person so church helps me a lot, yeah, to survive… A spiritual life, yeah, being with God.”* Participant from Focus group 3.

However, opportunities of going to church were not always guaranteed as some participants were requested by their employers to work over the weekend.

*“So whatever is going on I just keep it to myself and the only thing I do is to go to church every week-end. I’m asking them, please don’t make me work on Sunday because this is the only time I can go and do something for myself.”* Participant from Focus group 2.

The participants’ narratives suggested that their social support networks were limited. Only two participants in this study identified their former employers as a source of social support. In one case, it was a reciprocal relationship in which the participant was providing emotional support and advice to the former employer who were faced with challenges of parenting two children living with autism.

### Mental health, resilience and access to care

To enhance our understanding of the participants’ experience of mental health challenges and their resilience, we first explored their perspectives about mental health and illness.

#### Perspectives on mental health

When we asked the participants what the term mental health meant to them, the following responses emerged: *“healthy environment”, “happy thinking”, “finding joy everyday”, “ability to manage your stress”, “ability to control your emotions”, and “capability to cope with things”.* Overall the participants felt that mental health is related to one’s ability to control one’s emotions, cope with stress and make rational decisions. Several indicated that their mental health was at jeopardy due to their living and working conditions. However, they also stressed that they had to learn to control and avoid showing their emotions in order to come across as a competent caregiver.

*“I can’t go depressed. I cannot show like I have bad day. Like I learn how to control myself. Before in my country I will tell you in your eyes how I feel but because of this program I do not know if I learned something or I’m good actress… I never show people how bad I feel...I will control my emotions.”* Participant from Focus group 2.

Some felt that showing any sign of depression or sickness might cause them to lose their jobs. They were put into a position whereby they had to acknowledge and empathise with their employers’ emotions and problems but had to hide their own.

*“They don’t want a depressed person to take care of their children. They do not want to hear your problems. Their problem is very important. If someone dies in their families it’s important. If you have a problem or someone dies you have to say everything is fine and leave your problems in the basement and you have to go and you have to be happy to take care of the children”.* Participant from Focus group 3

#### Perspectives on mental illness

When we asked the participants what the term mental illness meant to them, the following terms were used: “*crazy”, “being insane”, “cooko”, “aggressive”, “mad”, and “nervous breakdown”*. For the participants, mental illness was a sign of weakness and inability to control one’s emotions and cope with life stressors. Their answers and views of mental illness reflected their socialized beliefs.

*“Back home and even here also because it’s a common idea that if you are mentally unhealthy you are insane. But come to think of it, it’s not just that because there are times in our lives when we feel depressed and I think it’s a matter of how you cope with those stresses you encounter each day. It’s just how you deal with it. If you are strong enough, mentally strong enough, you will cope but if you are not that strong then you will go [into] depression and other mental problems in that matter”.* Participant from Focus group 2.

The majority of participants considered depression, anxiety, and stress as signs of mental illness but they believed that people’s strength to cope is the main determinant of mental illness. In other words, although most of participants expressed feeling depressed, anxious and stressed since their arrival to Canada they felt they were strong enough to control and overcome those emotions.

“*Filipinos by nature are resilient, you can prove to the world that Filipinos are resilient”. “I feel strong and I thank God for it ... I do not want to be depressed but I want to be strong for my son”.* Participant from Focus group 3.

Perhaps it was the desire for a bright future for their children and families that kept them resilient. Many shared with each other strategies that had helped them endure their harsh realities -- turning to a higher power by going to church to pray; reducing stress by taking walks, listening to music, and singing; maintaining connections by talking to family or friends via Skype or phone; and releasing frustration by screaming and pulling out grass from the backyard when they were alone. Most of all, they identified the hope of gaining permanent residency and family reunification upon their completion of the 24 months of required caregiving work as the driving force behind their resilience.

#### Access to mental health services

With respect to using health services in Canada, participants indicated that they had seen family doctors, gone to walk-in clinics or to hospitals for a variety of ailments that they partly attributed to their working conditions. They also identified several challenges that they had encountered when seeking health care; a common challenge was the mandatory 3 months waiting time for new immigrants to access health care. One participant stated “My aunt said don’t be sick for the next three months”.

The focus group discussion also revealed that many participants neither used existing mental health services nor knew what services were available to them. In addition, their attitudes towards using mental health services were also influenced by their cultural beliefs and values. Many stated that they were not used to seeing psychiatrists, as this would be a sign of weakness and inability to cope with one’s problems.

*“In the Philippine, if we go to psychiatrists they think you are crazy.”* Participant from Focus group 3.

“*If I am depressed or anxious it is my decision to overcome this thing.”* Participant from Focus group 2.

Furthermore, trust and faith played a strong role in how the participants deal with mental health problems. They were more inclined to either talk to friends or family, particularly someone who they trusted or who had experienced a similar ordeal, in addition to praying to God.

“*As long as we have someone to pour our hearts out with them, all our anguish, our pain will disappear. And we leave our needs and our longing to God”.* Participant from Focus group 2.

*“When you tell your problems to someone that you know or that you’re familiar [with] it’s better than going to a clinic or something that you don’t know. So we better share our stories to someone we trust.”* Participant from Focus group 1.

Only one participant indicated that she had used a counsellor/psychotherapist and that was because she did not have anyone else to discuss her situation with.

*“I was depressed at times so I really talked with a counsellor at a walk-in clinic near my home. I said I am really stressed out. I don’t know how to get over [that] because financially my mom, sister and daughter and all siblings are depending on me. And I do not know how to get over it and I said I need someone to talk to. So I had a counsellor…So I had once”*. Participant from Focus group 1.

The participants’ narratives suggested that their access to culturally relevant and inclusive mental health information and resources was limited.

## Discussion

Our study examined migrant caregivers’ experience of working and living in Canada and how these experiences affected their mental health, a subject that to-date has been understudied. The study found that while these workers encountered challenges similar to other TFW groups such as migrant farm workers and other immigrant groups, they also experienced many unique challenges based on the contexts of their gender and the type of work they did. These unique challenges affected their mental health and majority complained of being depressed, anxious, and felt isolated.

By virtue of working and living in the confined space within their employers’ homes, many had lost the protective boundary between work and after-work time and space. Although employers of live-in caregivers are required by law to provide housing that meet municipal building codes and regulations, many participants described living in substandard and unsafe conditions that they did not feel being “at home.” Their circumstances reflected the phenomenon of “housed but homeless,” [33] as they did not have the autonomy to create a home within the assigned space. Psychologists and sociologists have proposed that the home is not merely a space of shelter or residence. Home is a place with special qualities such as privacy, personal identity, self-expression and a sense of belonging; it is “a unique place where a person’s past, present, and future selves are reflected and come to life” ([34], p. 347). For many participants in this study, their living space was a bed to sleep in after they finished a long day of work. They did not have the power or autonomy to set boundaries with their employers to secure privacy and free time from the children or additional unpaid work. Many reported substandard living conditions, long working hours that sometimes included the weekends, being asked to do “domestic” work beyond caregiving, uncompensated overtime work, and disrespect and abuse from employers and sometimes even the children they cared for. These results corroborate those from other studies of TFWs [8–11, 35].

Participants also expressed concerns about a decline in their general health since coming to Canada. Several studies have demonstrated a “healthy immigrant effect” among TFWs and other immigrants [17, 19, 20]. The narratives of the participants suggested that their precarious work and migration status had imposed tremendous mental health stress on them. They constantly lived in fear about losing their jobs and the impossibility to find a replacement job. They worried about the harsh reality of being deported and losing their livelihood, not only for themselves but also their families back home, who relied on their financial support. This constant fear and the lack of labour rights protection put them at the whim of their employers. Indeed, the migrant caregivers’ legal rights and social safety were compromised by the Canadian government’s managed migration policy of a completion of 24 months of caregiving work as a requirement to apply for permanent residence. Under this immigration requirement, the employers were given more implicit power to violate their agreements to provide fair, safe, and ethical working conditions because they are aware of the temporary migrant caregivers’ desperation for permanent residency and family reunification. As a result, many migration caregivers, as indicated by our participants, experience prolonged sense of despair, alienation and powerlessness, which compromise their mental health. This finding is consistent with other studies on the effects of financial and job-related difficulties, inadequate living and working conditions, and discrimination on mental health [14, 17, 36–39]. Furthermore, diasporic experiences such as losing one’s social networks, declined social status, underemployment and substandard living environment have also been identified as contributing factors to immigrants’ experiences of depressive moods [40, 41].

Social support and community connectedness or sense of belonging have been reported as protective factors against stress and for sustaining emotional, mental and physical health [42–45]. Our study results showed that many migrant caregivers have limited access to social support. Most participants identified their families back home, new friends in Canada, and faith groups as their key sources of social support. Trust was also identified as a major factor behind their decision about whom they would seek help from or confide in. However, getting support from their families was not always a viable option for many. Some avoided telling their families about the dehumanizing working and living conditions they were in because they did not want to burden burdening their families with stress and despair, and also because they felt that their back home were just as powerless as they were in these situations.

Structural inequalities persistently create, reinforce and perpetuate the positioning of migrant live-in caregivers and other TFW as the “Other” who are deemed lesser and therefore do not need to be treated equally or fairly. “Othering” is a process in which the dominant group sets up its own identity apart from the “Other”. People being “Othered” experience this domination in the forms of physical, social, psychological and economic disempowerment and exclusion [46]. In the case of migrant live-in caregivers, Othering took place in myriad ways, including the systematic practice of deskilling of professionally trained migration workers (e.g., teachers and nurses hired as caregivers and domestic workers); hidden practices of employment standard violation (e.g., unrestricted on-call work demands, substandard housing, workplace abuse); and dehumanizing practices of deprivation (e.g., missing significant life events such as caring for dying loved ones, birthdays and graduations of children, funerals, etc.). The detrimental effects of Othering on migrant workers, manifested as social alienation, loss of positive identity, displacement and dehumanizing experiences, were similar to those found in other studies among refugees, aging ethnic seniors, and people living with chronical health problems and mental illness [47–50].

The World Health Organization (WHO) defines mental health as “a state of well-being in which every individual realizes his or her own potential, can cope with the normal stresses of life, can work productively and fruitfully, and is able to make a contribution to her or his community” ([51], p.1). In this study, the participants’ definition of mental health mainly focused on *being in control of one’s emotion* and *the ability to cope with stress*. It seems that their definition is influenced by two main factors: a) their working-living conditions and their subordinate, precarious position in Canada, and b) their socialized beliefs and values. Some of the migrant caregivers described their everyday experiences as being like present-day slaves, having to suppress their feelings of frustration, rage, despair, anxiety, and powerlessness in order to avoid punishment from their employers, which in their case was the fear of being dismissed or deported. Scott [52] asserts that self-control/self-restraint is a learned attribute of subordinate groups “because their vulnerability has rarely permitted them the luxury of direct confrontation” (p.136). Being aware of the precarious nature of their employment, migrant caregivers are reluctant to show any signs of depression, stress, anxiety or emotional problems in front of their employers or seek medical care for fear of losing their employment. As described earlier, Canada’s TFW Program only admits workers who are deemed to be in excellent health. Our study results also suggested that the participants’ socialized values and beliefs hold mental illness as an abnormality tainted with stigma. This corroborated previous findings which revealed high level of stigma espoused by cultural and religious values among Filipino in seeking mental health [53, 54]. However, our study also showed that participants expressed a persistent emphasis on “self-control of emotions” when discussing mental health. This heightened sense of self-control could possibly be related to their fear of deportation and medical inadmissibility when they apply for permanent residence in Canada, which subsequently hindered their motivation in accessing health services.

The narratives of the participants showed that their working-living circumstances had steered them into living “double” lives: a public life in which they must exhibit cheerful dispositions and positive emotions, and conceal their suffering, frustration, anxiety and depression (as expected by their employers and others); and a private life in which they cried themselves to sleep, screamed from the top of their lungs when their employers were not present, and prayed for obliteration of the insufferable challenges that they faced in a foreign land. Their everyday reality resembles what Scott [52] refers to as public versus private and hidden transcripts. Public transcript is the open interaction or expression on public stage between subordinates and those who dominate, whereas hidden transcript refers to the discourse that takes place “off stage” when subordinates are safe from the surveillance of powerholders. Unequal power relations are indicated by a vast discrepancy between public and private transcripts (Scott, [52]). In other words, public transcript is the way people learn to respond, act or behave in a power-laden situation which may be totally different from or opposite to what they really think, talk, act or behave in private- in the presence of those whom they could voice their unspoken thoughts. This dichotomy is well demonstrated in the migrant caregivers’ discussion of their lives in Canada. They indicated that they had to act differently in front of their employers to cover any signs of displeasure with their current situations; they also shared how hard they try to rely on their own inner strengths to cover the feelings of loneliness, detachment, isolation, pain and insult which they sustain. Furthermore, their working-living circumstances had placed them between a rock and a hard place, whereby they had to project their public transcript not only in front of their employers but also their families and loved ones back home because of their sense of shame about their declined social status and fear of disappointing their families. Our study also showed the divergence of the participants’ public and hidden transcripts and their impression management. When the participants suppressed their feelings from the direct observation of the power holders (employers and Canadian government), they experienced immense suffering and mental anguishes. However their hope of becoming permanent residents in Canada and subsequently achieving family reunification seemed to have become their source of strength and determination to survive. Their high survival skills might be espoused by the economic constraints that they had endured in their respective home countries, and their desire to provide a better life for themselves and their families in Canada.

Some policy changes have been made to the TFW- Caregiver Program in recent years but they do not seem to go far enough to address issues identified in this study. Considerable work still needs to be done to improve the working environment and empowering workers to negotiate with their employers, and ultimately ensure better physical and mental health for this vulnerable group. Policy changes based on human rights and social justice are required to improve the economic and social integration of TFWs; these changes must include the enforcement of employment and labour standards, with adequate inspection and monitoring of the migrant caregivers’ living and working conditions and accountable consequences for employers who violate the regulations. Since labour standards are under provincial and territorial jurisdictions, collaboration across different levels of government and departments is critical.

Although migrant caregivers are no longer required to be live-in caregivers, many do not earn adequate salaries that enable them to afford housing and transportation to their workplace. In this study, 73% of our participants earned less than $1999 per month and with remittance to family, few had any extra money to pay for independent housing in the Greater Toronto Area – one of the most expensive cities in Canada. Thus, policy changes must ensure adequate financial compensation and housing subsidies that enable caregivers to live independently outside their employer’s residence. They could also draw from current employment standards established for Canadian workers in similar occupations (e.g., childcare, homecare, personal support, etc.). In other words, the Canadian government must narrow the huge gap between the human rights and labour protection given to Canadian workers and foreign migrant workers.

To reduce exploitation and abuse of migrant caregivers, the Canadian government must also change its “managed immigration” policy to remove the required completion of 24-month service and allow migrant caregivers to apply for permanent residence upon arrival in Canada. This would reduce migrant caregivers’ experience of social isolation and prolonged separation from their families and loved ones. In addition, efforts should be made to leverage social networks and connection to faith organizations currently used by the caregivers. Increasing opportunities for migrant caregivers to engage with organizations within their ethno-cultural communities may also help reduce isolation. This could be coupled with access to taking part in other cultural and mainstream organizations to facilitate integration into the broader Canadian society. Increased funding should be provided to community organizations that reach out to caregivers and other TFWs. Utilizing other methods of social interaction e.g. online meetings may enhance participation based on the migrant caregivers’ limited free time and work demands.

Given that most of our participants lacked information about their employment rights, healthcare systems, mental health resources, and community support, it is important to tap into (e.g., ethno-specific organizations, faith organizations, and the Internet) and engage these organizations in the provision of health and legal information and resources. Organizations currently providing mental health services must consider the unique needs of migration caregivers and other TFW and provide culturally relevant and inclusive programming and services. Our recommendations have been espoused by many advocates and researchers who have identified the many shortcomings of the current TFW regulations [5, 8, 10, 17, 18].

A couple of considerations have to be kept in mind when interpreting the results of this study. Our study participants mainly included a specific subgroup of TFWs (i.e., live-in caregivers) and the majority were from the Philippines. Also, since most of the women in this study were relatively well educated and they could all speak English, we might not have captured the issues and barriers experienced by other migrant caregivers or TFWs who are less educated and who cannot speak English well.

While our study results cannot be generalized to the larger TFW population, the insights gained from this study could be applied to TFWs in similar contexts; many of the issues identified in this study are likely relevant to other TFW groups who work and live in similar conditions. Since the Filipino community constitutes one of the largest sources of live-in caregivers [55, 56], the findings in this study provide valuable insights to support collective action and empowerment to promote resilience within this vulnerable subpopulation.

## Conclusion

This study has generated the much needed new knowledge about the mental health of TFWs in Canada, particularly those in the Caregiver Program, and how their living and working conditions shape their mental health. This knowledge is critical and can be used to inform policy changes as well as programming and services to ensure optimum health outcomes for TFWs. For more than five decades, Canadians have benefitted immensely from the hidden exploitation of migrant workers in many economic sectors. As researchers, it is critical that we make these inequities visible to promote open dialogue and collectively develop new labour and immigration policies that uphold the human rights and dignity of migrant workers from low-income countries.
